# Effect of Hydrophilic/Hydrophobic Nanostructured TiO_2_ on Space Charge and Breakdown Properties of Polypropylene

**DOI:** 10.3390/polym14142762

**Published:** 2022-07-06

**Authors:** Jun-Guo Gao, Hong-Shuo Liu, Ting-Tai Lee, Uwe Schachtely, Hitoshi Kobayashi, Li-Li Li

**Affiliations:** 1 Key Laboratory of Engineering Dielectrics and Its Application, Ministry of Education, Harbin University of Science and Technology, Harbin 150080, China; hasaiatong@163.com (H.-S.L.); lily_li@hrbust.edu.cn (L.-L.L.); 2 Evonik Specialty Chemicals (Shanghai) Co., Ltd., Shanghai 201108, China; tingtai.lee@evonik.com; 3 Evonik Operations GmbH, Hanau-Wolfgang, 63457 Essen, Germany; uwe.schachtely@evonik.com; 4 Evonik (SEA) Pte Ltd., Singapore 138567, Singapore; hitoshi.kobayashi@evonik.com

**Keywords:** polypropylene, blend insulation, crystallization conditions, space charge, AC/DC breakdown

## Abstract

Polypropylene (PP) has received more and more attention in the field of insulating materials as a recyclable thermoplastic. To further enhance the applicability of polypropylene in the field of insulation, it needs to be modified to improve its electrical properties. In this paper, the impact mechanism of AEROXIDE^®^ TiO_2_ P 90 (P90) and AEROXIDE^®^ TiO_2_ NKT 90 (NKT90) as nanosized hydrophilic and hydrophobic fumed titania from Evonik on the electrical properties of PP was studied mainly through the crystallization behavior and space charge distribution of PP nanocomposites. Two kinds of nanostructured TiO_2_ were melt-blended with PP according to four types of contents. The results of alternating current (AC)/direct current (DC) breakdown field strength of the two materials were explained by studying the microstructure and space charge characteristics of the nanocomposites. Among them, hydrophilic nanostructured TiO_2_ are agglomerated when the content is low. The spherulite size of the nanocomposite is large, the space charge suppression ability is poor, the charge is easy to penetrate into the pattern, and the AC/DC breakdown field strength is significantly reduced. However, hydrophobic nanostructured TiO_2_ has better dispersion in PP, smaller spherulites, more regular arrangement, and less space charge accumulation. The charge penetration occurs only when the nanostructured material content is 2 wt%, and the AC/DC breakdown strength increases by 20.8% at the highest when the nanostructured material content is 1 wt%. It provides the possibility to prepare recyclable high-performance DC PP composite insulating materials.

## 1. Introduction

The increasing demand for electric energy has brought more severe challenges to the infrastructure construction of electric power systems. In the current field of high-voltage cables, the polymer insulation material is widely used for its low density, low dielectric constant, low dielectric loss, good mechanical flexibility, and convenient processing [1,2,3,4]. Cross-linked polyethylene (XLPE) is a widely used cable insulation material in this field, which has been deeply studied by engineering scholars at present [5,6,7]. However, with the gradual improvement of power transmission requirements and the proposal of environmental protection concepts such as green development to the society, the application of XLPE is limited by its characteristics, and the exposed problems are gradually increasing [8,9]. Finding alternative materials has become the focus of attention now.

Polypropylene (PP) is a kind of environment-friendly and recyclable thermoplastic material with excellent heat resistance [10,11,12]. As an insulating material, it has a smaller relative density, does not need cross-linking, has high electric breakdown strength, and has a strong ability to suppress space charge [13,14,15]. Overall, PP is superior to polyethylene (PE) in most aspects, and its development prospect in the cable industry is also very considerable. However, some properties of polypropylene still have room for improvement.

To further improve the physical properties and electrical properties of PP, scholars have tried the method of doping nanostructured material. For example, adding nanostructured SiO_2_, rattan, *Carpinus betulus* L. (CB) powder, and corn maize flour into PP can enhance the physical properties of the material, such as toughness and heat resistance [16,17,18,19]. The others focused on electrical properties. Phichet Ketsamee studied the effect of surface-modified nanostructured MgO on the breakdown strength of PP composites; after treating the surface of nanostructured MgO with 3-aminopropyl triethoxy, the DC electrical breakdown strength increased by 16% [20]. The effect of polyamide/clay nanocomposites (NCs) on the comprehensive electrical properties of PP was investigated by Fuse [21]. The space charge characteristics and DC electrical breakdown strength of PP/MgO composites were studied by Zhou Yao [22]. Polyolefin elastomer (POE)/ZnO/PP modified composites were made by Xu Hang, and the crystalline state, space charge characteristics, and DC electrical breakdown strength of the composites were studied. The results showed that ZnO nanostructured particles could promote the crystallization of the composites, reduce the space charge accumulation, and effectively improve the electrical breakdown strength of the composites [23].

There are also some studies using TiO_2_ as a nanofiller. For example, the effects of surface-treated MgO, TiO_2_, ZnO, and Al_2_O_3_ on the dielectric properties, DC volume resistivity, space charge behavior, and DC electrical breakdown strength of PP composites have been researched by Yao Zhou [24]. The effect of nanostructured Al_2_O_3_, TiO_2_, and organoclay on the electrical properties of PP/Ethylene Propylene Diene Monomer (EPDM) was investigated by Muhammad Safwan Hamzah; 2 vol% Al_2_O_3_ and TiO_2_ were found to improve the electrical breakdown voltage of PP/EPDM [25].

Overall, there have been many studies on the modification of PP by doping nanostructured materials, and the problems of space charge accumulation and electrical breakdown strength have been analyzed. However, the nanostructured materials were all surface-treated, and no comparative experiments have been carried out. Even though nanostructured TiO_2_ is used as a filler, the electric shock strength and space charge distribution of the composites have not been studied deeply enough. As a non-toxic inorganic filler, TiO_2_ has good chemical stability, ultraviolet (UV) filtering ability, and low cost [26,27,28]. Some studies have also proved that TiO_2_ can improve the tensile strength of the matrix [29,30]. Therefore, it is of interest to comparatively analyze the mechanism of the effect of surface-treated and unsurfaced nanostructured TiO_2_ on the space charge accumulation and electric breakdown strength properties of PP.

In this paper, nanoscale hydrophilic and hydrophobic fumed titanium dioxides from Evonik’s AEROXIDE^®^ TiO_2_ P 90 (P90) and AEROXIDE^®^ TiO_2_ NKT 90 (NKT90) were added to PP in varying amounts. The nanocomposite film was prepared by melt-blending and hot-pressing methods. Its microstructure was characterized by a scanning electron microscope (SEM), polarized light microscope (PLM), and X-Ray Diffractometer (XRD). The change in the electrical properties of the nanocomposite is reflected by the space charge distribution and the AC/DC electrical breakdown strength. The effects of nanoparticles on the electrical properties of polypropylene were compared and analyzed.

## 2. Materials and Methods

### 2.1. Materials

**Figure 1:**
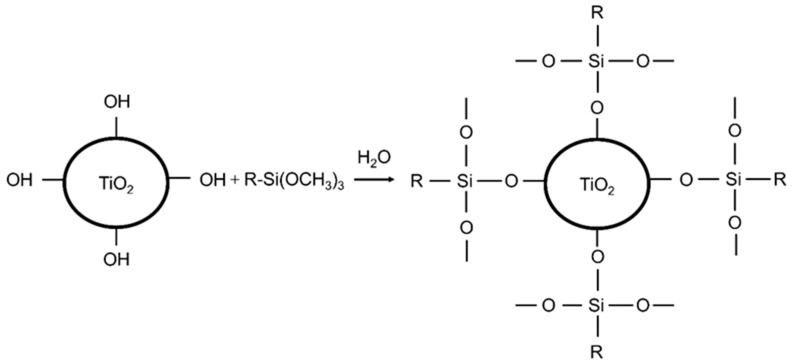
Alkane modification of nanostructured TiO_2_ powder.

For the convenience of directly observing the effect of nanostructured TiO_2_ on the dielectric properties of PP, isotactic PP cable compound (4874, Borealis AG, Vienna, Austria) with a melt flow index of 2.8 g/10 min (230 °C 10 kg) and tensile strength of 55 MPa was used as the matrix material. Nanostructured TiO_2_ (NKT90/P90, Evonik Industries AG, Essen, Germany), is divided into P90 (hydrophilicity) without surface treatment and NKT90 (hydrophobicity) with alkylsilane surface treatment. Figure 1 is a schematic diagram of the reaction process of alkylsilane on the surface modification of nanostructured TiO_2_.

### 2.2. Sample Preparation

The PP and two kinds of nanostructured materials were placed in an oven and dried at 60 °C for 12 h before sample preparation. Then, they were added to the Torque Rheometer (RM-200A, Hapu electrical technology limited liability company, Harbin, China) according to four proportions of 0.5 wt%, 1 wt%, 1.5 wt%, and 2 wt%, respectively, and treated at 190 °C for 20 min, with a speed of 40 rpm [31].

The thin-film samples with different thicknesses were made by the Plate Vulcanizing Machine (XLB-350, Haimen Jinma Rubber & Plastic Machinery Technology Co., Ltd., Haimen, Jiangsu, China). The pressure was increased to 15 MPa within 20 min, and then the sample was cooled to room temperature at the same pressure and rate of cooling. The obtained samples were stored in vacuum bags to eliminate the influence of moisture. The size of the sample was a circle with a diameter of 8 cm, and a thickness of 50 μm, 200 μm, and 250 μm with a variation of ±10%. The abbreviations corresponding to nanocomposites in this paper are shown in Table 1. Among them, PP + 0.5 wt% P90 means nanostructured P90 particles doped with 0.5 wt% in PP, and so on.

### 2.3. Experimental Methods

The microstructure and nanostructured material dispersion of nanocomposites were studied by Ultra-high Resolution Cold Field Emission Scanning Electron Microscope (SEM, SU8020, Hitachi Construction Machinery Co., Ltd., Tokyo, Japan) at an accelerating voltage of 10.0 kV. Observation resolution was ≥2.0 nm. Before the experiment, we cut the sample and froze it in liquid nitrogen for 40 min to make it brittle. Subsequently, we sprayed gold on the fracture surface to avoid charge accumulation during the observation.

The crystallization degree of the sample was characterized by the Polarizing Microscope (PLM, LeicaDM2500, Leica Microsystems, Wetzlar, Germany). Since PP is a semi-crystalline polymer, at a specific temperature, it grows into a spherocrystal symmetrically around the crystal nucleus. Spherocrystal has optical anisotropy and can refract light. 

Polypropylene is a polymer with crystalline and amorphous regions. The crystal form of PP is influenced by crystallization conditions and additive filler. The typical crystal forms include α, β, and γ [32]. The change of crystal form directly changes the performance of the material. The crystal morphology of PP composites was analyzed by the X-Ray Diffractometer (XRD, Empyrean, PANalytical Corporation, Alemlo, The Netherlands). Cu target $K_{\alpha }$ radiation ($K_{\alpha }=1.54$ Å), excitation voltage 40 kV, excitation current 40 mA, scanning range 2θ = 10°~30°, scanning speed 3°/min, and angular resolution 0.026° FWHM on LaB6. The test sample was a thin slice with a thickness of 0.2 mm. Multiple tests were conducted for each sample by repeating the experimental results.

A pulsed electroacoustic space charge test system (PEA, Shanghai Jiao Tong University, Shanghai, China) with a pulse width of 5 ns was used to study the space charge distribution [33]. The thickness of the sample was about 250 μm. A 40 kV/mm direct current (DC) was applied to the sample for 30 min and then short-circuited for 30 min while measuring. The upper electrode of the PEA measurement system is semi-conductive, the lower electrode is aluminum, and silicone oil is used as a medical ultrasonic couplant.

The sample was placed into the drying oven at 60 °C for 10 h before the AC/DC electrical breakdown strength test to exclude the effect of water. To prevent surface flashover, the whole process was carried out in an electrical breakdown box immersed in transformer oil (45#, Sinopec Lubricant Co., Ltd., Wuhan, China) at room temperature (around 30 °C). The voltage boosting speed of AC is 1 kV/s, and DC is 2 kV/s. The diameter of the cylindrical electrode was 25 mm. Fifteen electrical breakdown voltages were recorded for each sample, and the measurement was better than 0.1%. After calculating the electrical breakdown strength, the test data were processed by the Weibull distribution method [34].

## 3. Results and Discussion of Simulation

### 3.1. SEM Analysis

**Figure 2:**
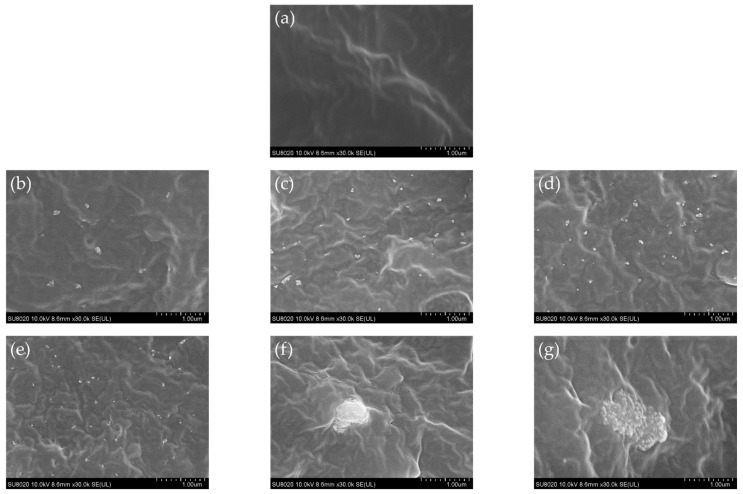
SEM images of PP nanocomposites with different NKT90/P90 contents. (**a**) PP; (**b**) PP + 0.5%NKT90; (**c**) PP + 1%NKT90; (**d**) PP + 1.5%NKT90; (**e**) PP + 2%NKT90; (**f**) PP + 0.5%P90; (**g**) PP + 2%P90.

The brittle fracture section of TiO_2_ nanocomposites obtained by SEM is shown in Figure 2.

The dispersion of nanoparticles in PP was analyzed by ImageJ. The measured average nearest neighbor distance of 0.5NKT90 was 0.5 μm, 1.0NKT90 was 0.12 μm, 1.5NKT90 was 0.11 μm, 2.0NKT90 was 0.05 μm. The size of the agglomerates in Figure 2f is about 0.6 μm × 0.8 μm, and the size of the agglomerates in Figure 2g is about 1.3 μm × 0.7 μm. It is proved that the distance between nanoparticles decreases gradually with the increase of nanoparticle content.

As can be seen, the nanostructured NKT90 can maintain good dispersibility in a PP matrix and hardly agglomerate, and with the increase of nanoparticle content, nanostructured material will overlap. However, when the content of nanostructured P90 is 0.5 wt%, the agglomeration occurs, and when the content is 2 wt%, there is a more significant agglomeration. From SEM, it can be seen directly that hydrophobic nanostructured TiO_2_ is well dispersed in PP, while hydrophilic nanostructured TiO_2_ will agglomerate in PP. The surface of hydrophilic TiO_2_ particles contains a large number of hydrophilic groups—OH, and the dehydration or condensation between Si–OH in alkylsilane and hydroxyl Ti–OH on the surface of TiO_2_ particles neutralizes the hydrophilic group −OH. Therefore, the hydrophobic TiO_2_ particles will have better dispersibility. The interfacial effects of nanostructured materials are related to their dispersibility. When the dispersion is not good, it will cause defects inside the material, distorting the electric field and reducing the dielectric properties of the nanocomposite [35,36,37].

### 3.2. PLM Morphology

**Figure 3:**
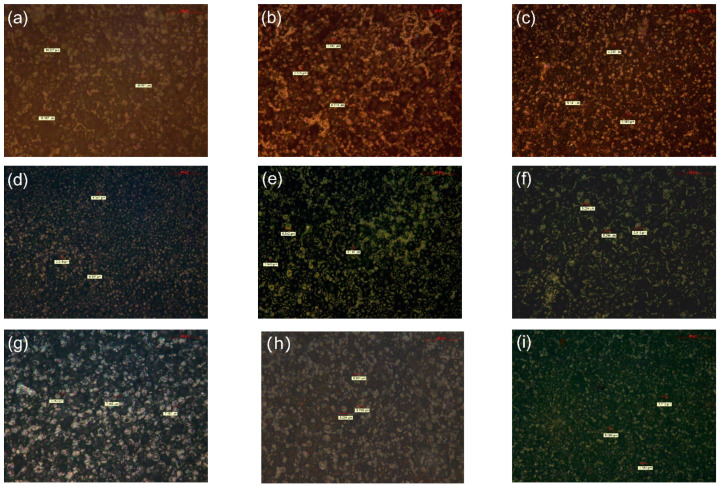
PLM results of polypropylene nanocomposites with different amounts of NKT90/P90. (**a**) PP; (**b**) 0.5NKT90; (**c**) 1.0NKT90; (**d**) 1.5NKT90; (**e**) 2.0NKT90; (**f**) 0.5P90; (**g**) 1.0P90; (**h**) 1.5P90; (**i**) 2.0P90.

Figure 3 is the PLM result of TiO_2_/PP nanocomposites. As can be seen, after adding these two kinds of nanostructured materials, the crystal size of the composite decreased, and the spherulite size of the NKT90 nanocomposite clearly decreased. The size of 1.0NKT90 was smaller, and the arrangement was more orderly, while the regularity of 2.0NKT90 was worse, and the uniformity of size was also reduced.

The above phenomenon can be explained as follows: with the low content of nanostructured material, the structure of the PP molecular chain segment will be changed, the molecular chain will be chemically degraded, and the spherulite size will decrease. However, with the increase of nanostructure, the content of peroxide radicals also increases, which leads to the possibility of branching of some chain segments [38]. At the same time, the homogeneity of the molecular chain will deteriorate, and the regularity will be reduced, which will eventually lead to the uneven size of the spherulites and the irregular arrangement.

The results of SEM show that the addition of nanostructured material in the composite will affect its crystallization, and good dispersity leads to a promising trend in the crystallization of the composite.

### 3.3. XRD Analysis

**Figure 4:**
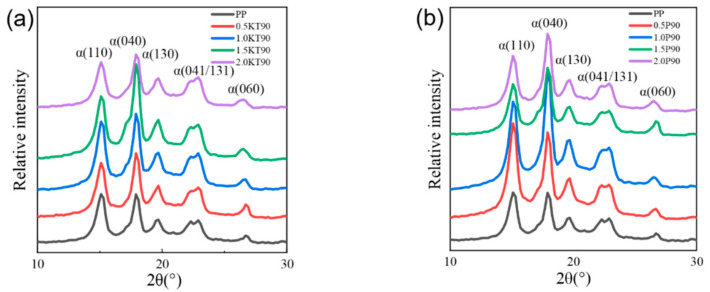
XRD curves of PP nanocomposites with different NKT90/P90 content. (**a**) Results of NKT90 nanocomposites. (**b**) Results of P90 nanocomposites.

The XRD test results of two TiO_2_/PP nanocomposites are shown in Figure 4. It can be seen that all nanocomposites had six obvious peaks, namely α (110), α (040), α (130), α (041), α (131), and α (060). There were no new diffraction peaks, which means that the addition of two nanostructured materials did not change the α crystal form of PP. Studies have shown that adding a nucleating agent to PP can change its crystalline morphology, thus affecting its electrical properties [39,40].

It can be observed that the intensity of the α (110) and α (040) diffraction peaks of PP was significantly enhanced after the addition of nanostructured materials, and the intensity of the other diffraction peaks also increased slightly. The overall diffraction peak intensity of the two nanocomposites first increased and then decreased with the content of nanostructured material, where the intensity of the α (110) diffraction peaks of 0.5P90 and 1.0NKT90 was the strongest, and the intensity of α (040) diffraction peaks of 1.0P90 and 1.5NKT90 was the strongest. As the intensity of the peaks in the same position is determined by the number of crystalline planes arranged in the same direction, a higher intensity of the peak indicates a higher degree of crystallization in the order of the crystalline plane, a faster nucleus growth rate at the same time and an increase in crystallinity [41].

It is explained that nanostructured TiO_2_ particles act as nucleators in PP, which increases the crystallinity and crystal rate of the material to a certain extent. Along with the increase of nanostructured material content, the branching of chain segments increased, the crystallization weakened, and the crystallinity decreased. It is consistent with the results observed by PLM [42].

The diffraction intensity of P90 nanocomposites is higher than NKT90. From the analysis of crystal growth rate, the reason may be that NKT90 has been treated on the surface, and the chemical bonds cause the growth rate of crystals around NKT90 to be lower than that of P90. From the analysis of crystal degree, PLM results show that in the same observation range, the spherulite size of P90 is generally more significant than that of NKT90, and the crystallinity of P90 is more extensive when there is little difference in spherulite distribution.

### 3.4. Space Charge Analysis

**Figure 5:**
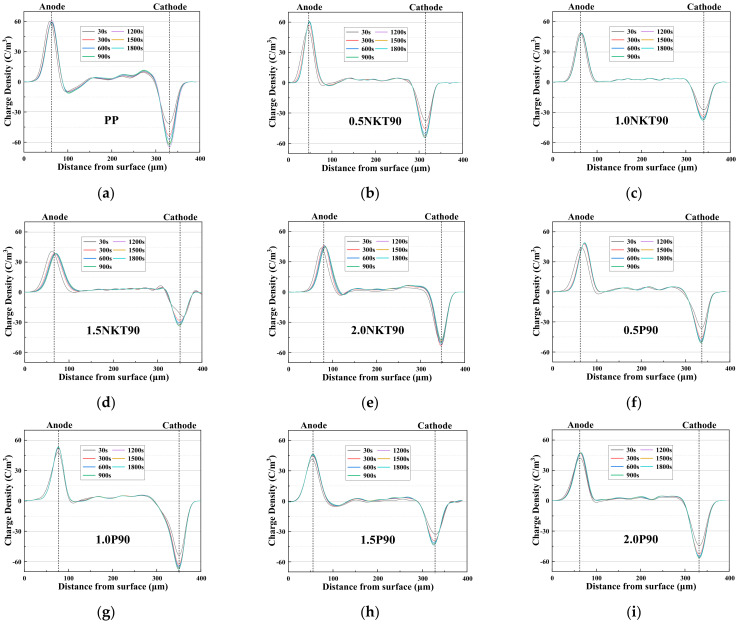
Charge distribution in the polarization phase of nanocomposites. (**a**) PP; (**b**) 0.5NKT90; (**c**) 1.0NKT90; (**d**) 1.5NKT90; (**e**) 2.0NKT90; (**f**) 0.5P90; (**g**) 1.0P90; (**h**) 1.5P90; (**i**) 2.0P90.

As for the high voltage direct current (HVDC) insulating materials, space charge accumulation is an essential parameter that affects the long-term properties and service life of insulation and equipment. Therefore, the space charge accumulation characteristics of two types of TiO_2_ nanoparticles with different contents in a polypropylene matrix were investigated by PEA. Figure 5 shows the distribution of space charge accumulation for different samples under the DC field of 40 kV/mm at room temperature for 30 min.

As shown in Figure 5, the anode of polypropylene starts to generate a heteropolar charge after the start of polarization. These heteropolar charges may be due to the ionization of impurities. The electric field strength between the electrode and PP is enhanced due to the heteropolar charge, and the charge is more easily injected into the PP at this time. Ions from the impurity ionization move slower; they are easily trapped by traps, resulting in charge accumulation [43]. Therefore, the density of space charges in PP increases with the increase of polarization time. 

It can be seen that the space charge accumulation was suppressed with the addition of nanomaterials from Figure 5. In the pressurized state, when the content of NKT90 was 0.5 wt%, 1 wt%, and 1.5 wt%, the accumulation of space charge was suppressed to a certain extent, and no heteropolar charge was generated at the initial stage of polarization. When the content of NKT90 reached 2 wt%, the inhibiting capacity of NKT90 to space charge began to decline, the internal charge density of the sample started to increase gradually, and the heteropolar charge was generated again near the anode. With the content of P90 being 1 wt%, the capacity of inhibiting space charge began to decline. From the previous SEM results, it was found that P90 is very poorly dispersed in PP. More nanostructured material creates more agglomerations and more defects, leaving more charge inside the nanocomposite.

**Figure 6:**
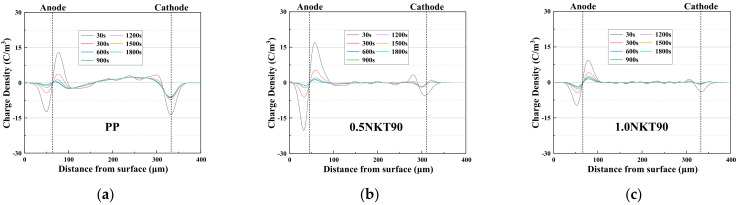

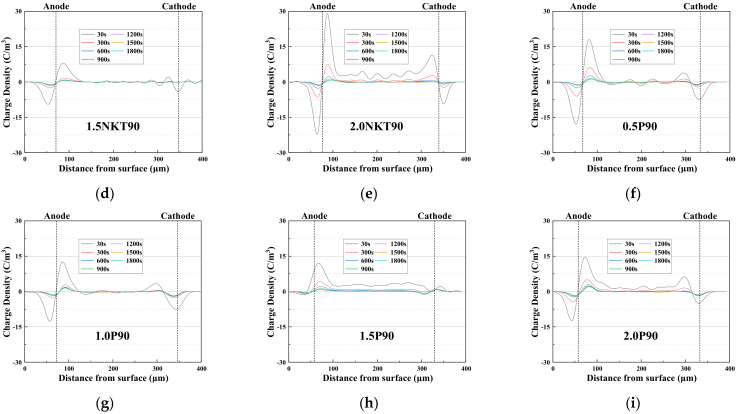
Charge distribution in the depolarization stage of nanocomposites. (**a**) PP; (**b**) 0.5NKT90; (**c**) 1.0NKT90; (**d**) 1.5NKT90; (**e**) 2.0NKT90; (**f**) 0.5P90; (**g**) 1.0P90; (**h**) 1.5P90; (**i**) 2.0P90.

The depolarization process is shown in Figure 6. When the content of NKT90 in PP was 0.5 wt%, 1 wt%, and 1.5 wt%, there was a small amount of residual charge in the sample. When the content of NKT90 was 2 wt%, and P90 content was 1.5 wt% and 2 wt%, there was obvious charge accumulation and uneven charge distribution inside the sample.

**Figure 7:**
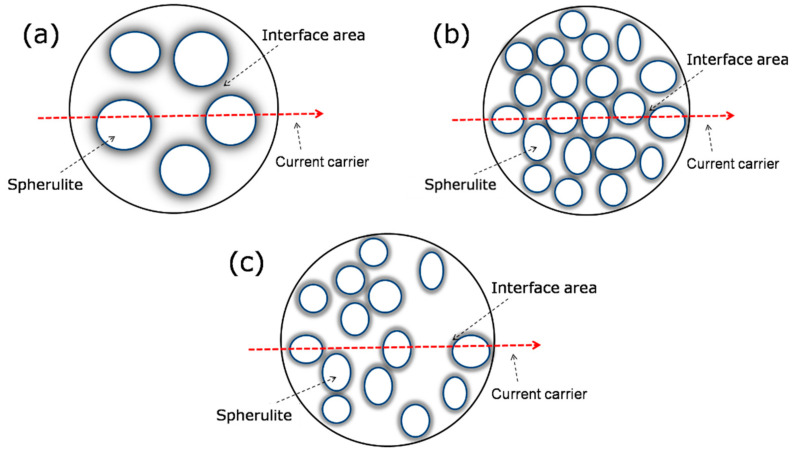
Charge motion under different crystallization conditions. (**a**) When the crystal size is large. (**b**) When the crystal size is small and the crystallinity is significant. (**c**) When the crystal size is small and the crystallinity is low.

For the depolarization process, the space charge inside the sample decayed continuously, and its rate had a great relationship with the apparent charge carrier mobility and trap depth [44]. Therefore, the situation that the space charge content increases first and then decreases with the addition of nanometer content can be analyzed from two aspects:

1. The spherical size of PP is large, and the crystallinity is low from the PLM test results. When the carrier moves in PP, as shown in Figure 7a, there are more amorphous regions and less interfacial regions, only some shallow traps in the amorphous region, and carriers can move easily in the composite material [45,46,47]. Thus, the residual charge density in the samples is larger. When a certain number of nanostructured NKT90 are mixed, the carrier motion is as in Figure 7b, such as 0.5NKT90, 1.0NKT90, and 1.5NKT90. At this time, the spherulite size decreases, and the crystallinity increases, which results in a large number of boundary regions. Deep traps exist in the boundary region between the spherulite and amorphous regions [45,46,47]. The number of deep traps increases, the movement of carriers is hindered, and the charge is captured as soon as it is injected into the sample. At this time, the residual charge in the specimen is less, such as 0.5NKT90, 1.0NKT90, and 1.5NKT90. As shown in Figure 7c, when excessive nanostructured material is mixed, the spherulite size decreases, but the crystallinity decreases. The area of the amorphous region with irregular spherulite distribution increases, and the blocking effect of composite materials on carriers begins to decline. The crystal size of 0.5P90, 1.0P90, and 1.5P90 is between PP and NKT90/PP nanocomposites, so its ability to suppress the movement of carriers is also between them.
2. The interfacial effect of nanoparticles is also one of the reasons for limiting the movement of carriers. The nanostructured TiO_2_ will generate an interfacial region with PP, and there are deep traps and shallow traps in the interfacial region [48,49]. When the content of NKT90 is low, the deep traps in the interfacial region can trap mobile charges, thereby inhibiting charge injection. As the nanostructured TiO_2_ (NKT90 or P90) spacing decreases or agglomerates, the interfacial region will overlap, which is conducive to the generation of low-resistance paths for easier carrier transport and a higher density of residual charge inside the sample, such as 2.0NKT90, 1.5P90, and 2.0P90.

### 3.5. Electrical Breakdown Strength Analysis

**Figure 8:**
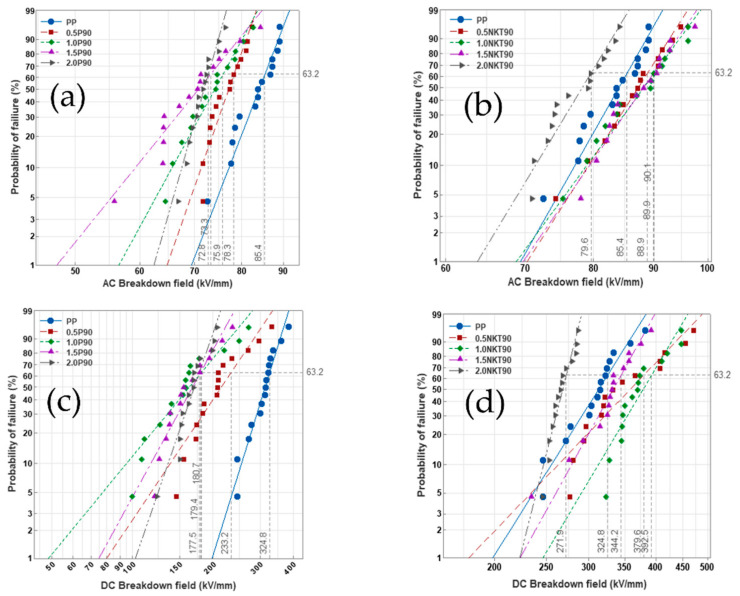
AC/DC electrical breakdown results of PP blends. (**a**) AC breakdown strength results of P90 composites; (**b**) AC breakdown strength results of NKT90 composites; (**c**) DC breakdown strength results of P90 composites; (**d**) DC breakdown strength results of NKT90 composites.

Figure 8 shows the Weibull-distributions of AC/DC electrical breakdown strength of PP/TiO_2_ nanocomposites with different nanostructured material content.

For the test of AC electrical breakdown strength, after adding P90, the AC breakdown strength of the sample visibly decreased. Currently, the AC breakdown strength of 0.5P90 is 78.73 kV/mm, which is 8.6% lower than that of PP. The AC electrical breakdown strength of 2.0P90 is 72.78 kV/mm, which is 14.8% lower than that of PP. After adding NKT90, the AC breakdown field strength of nanocomposites was not significantly improved, showing first a rising and then a falling. When the content was 1 wt%, the maximum electrical breakdown strength was 90.07 kV/mm, which is increased by 5.4%. When the content is 2 wt%, the lowest electrical breakdown strength is 79.61 kV/mm, a decrease of 6.7%.

When AC voltage is applied to the sample, most carriers injected into the electrode in the half-cycle are almost completely discharged in the second half cycle. Although a small number of carriers will be trapped in the deeper layer under the action of nanostructured material, this process will take a long time to observe the obvious charge accumulation, so nanostructured TiO_2_ has little effect on improving AC electrical breakdown strength. With the addition of P90, the decrease in field strength may be due to the poor dispersion of P90 in PP. Large-scale agglomeration is equivalent to defects in PP. With the increase of P90 and even the formation of visible particles, it is easy to cause partial discharge to reduce the insulation property of the materials.

Compared with the test results of DC electrical breakdown strength, the DC electrical breakdown strength of PP is 324.9 kV/mm. When the nanostructured material is P90, the DC electrical breakdown strength of nanocomposites with four proportions is lower than that of PP. The maximum breakdown strength is 233.2 kV/mm when P90 content is 0.5 wt%. The DC breakdown strength of nanocomposites with P90 content of 1 wt%, 1.5 wt%, and 2 wt% of P90 content basically ceased to change, with 180.7 kV/mm, 179.4 kV/mm, and 177.6 kV/mm, respectively. When the nanostructured material was NKT90, the DC breakdown strength of the nanocomposites with four proportions was higher than that of PP, which were 379.6 kV/mm, 392.5 kV/mm, 344.2 kV/mm, and 271.9 kV/mm, and showed a trend of increasing and then decreasing with increasing nanostructured material content. Among them, the breakdown strength of nanocomposites was the highest at 392.5 kV/mm when the content of NKT90 was 1 wt%, which is 20.8% higher than that of PP, and the lowest at 2 wt%, which is 16.3% lower than that of PP.

The DC electrical breakdown strength result is affected by the carrier concentration and carrier mobility in the medium; 0.5NKT90, 1.0NKT90, and 1.5NKT90 have less space charge accumulation, lower local carrier concentration, and relatively shallow charge injection depth. Compared with PP, the corresponding DC breakdown strength is improved. On the other hand, after the introduction of TiO_2_ nanomaterials, both the crystalline boundary region and the interfacial effect of nanoparticles hinder the movement of carriers, which reduces the movement rate of carriers, protects the molecular chain, prevents the generation of partial discharges, and ensures the insulation performance.

As the content of TiO_2_ nanomaterials increases, the probability of nanoparticles overlapping increases, the distance between them becomes shorter, and the effective travel of carrier migration increases. Currently, with the boundary region of the crystalline region and the interfacial effect of nanoparticles, carrier movement becomes easier. Excessive carrier migration speed will break the molecular chain of the polymer, resulting in lower DC breakdown strength [50]. From the Poisson equation [34], the more space charges accumulated inside the dielectric material, the more serious the electric field distortion, and the higher the local field strength will weaken the insulation performance of the material. However, because P90 is easy to agglomerate, it has a low barrier effect on carriers. As a physical defect, large-scale agglomeration greatly reduces the DC insulation performance of the material.

## 4. Conclusions

In this study, two kinds of nanocomposites with different contents of nanostructured TiO_2_ (hydrophilic P90 and hydrophobic NKT90) were made by melt-blending. The microstructure, crystallization, space charge distribution, and AC/DC breakdown strength of nanocomposites were studied. The effects of hydrophilic and hydrophobic nanostructured TiO_2_ on the electrical properties of nanocomposites were discussed by corresponding the microstructure changes to the macroscopic electrical behaviors.

1. P90 has poor dispersibility in PP and is easy to agglomerate, and its nanocomposite crystals are larger; NKT90 is well dispersed in PP4874, and the crystal size of the corresponding nanocomposites is smaller and more regular. With the increase of its content, the agglomeration of nanostructured gradually increases, and the crystallization becomes more nonsequenced. The addition of TiO_2_ did not change the crystal form of PP4874, indicating that the electrical properties of PP are changed by the growth rate and crystallinity of the crystal nucleus.
2. Nanostructured material can inhibit the space charge accumulation of the PP4874 to a certain extent. With the increase of nanostructured material content, charge accumulation will occur. The hydrophobic TiO_2_ is more effective in suppressing space charge.
3. When the content of NKT90 is 0.5 wt%–1 wt%, its DC breakdown strength is clearly increased, and its AC breakdown strength is also improved but not obvious enough. With the increase of nanostructured material content, the AC and DC breakdown strength gradually decreases. The overall AC/DC breakdown of P90 is lower than that of PP4874 due to agglomeration in PP4874.

According to comprehensive experiments, hydrophobic NKT90 is more suitable as nanostructured filling material, and its electrical properties are better when its content is 0.5 wt%–1 wt%.

The conclusions of this work will help to better understand the mechanism of the influence of hydrophobic and hydrophilic nanostructured TiO_2_ on the electrical properties of PP. Nanostructured TiO_2_ shows great potential in regulating the electrical properties of PP. It lays the foundation for related industries to make high-performance DC insulating materials with PP as the matrix.

## Figures and Tables

**Table 1:** Abbreviations for Borealis polypropylene 4874 and nanocomposites with 0.5 wt%, 1.0 wt%, 1.5 wt%, 2.0 wt% of P90 and NKT90 added, respectively.

Nanocomposites	Abbreviation
PP4874	PP
PP + 0.5 wt% P90	0.5P90
PP + 1.0 wt% P90	1.0P90
PP + 1.5 wt% P90	1.5P90
PP + 2.0 wt% P90	2.0P90
PP + 0.5 wt% NKT90	0.5NKT90
PP + 1.0 wt% NKT90	1.0NKT90
PP + 1.5 wt% NKT90	1.5NKT90
PP + 2.0 wt% NKT90	2.0NKT90

## Data Availability

The data presented in this study are available on request from the corresponding author.
